# Supplementary figures and images for: Inorganic sulfur fixation via a new homocysteine synthase allows yeast cells to cooperatively compensate for methionine auxotrophy
Source: PLoS Biol. 2022 Dec 1;20(12):e3001912. doi: 10.1371/journal.pbio.3001912 (PMC9757880; doi:10.1371/journal.pbio.3001912)

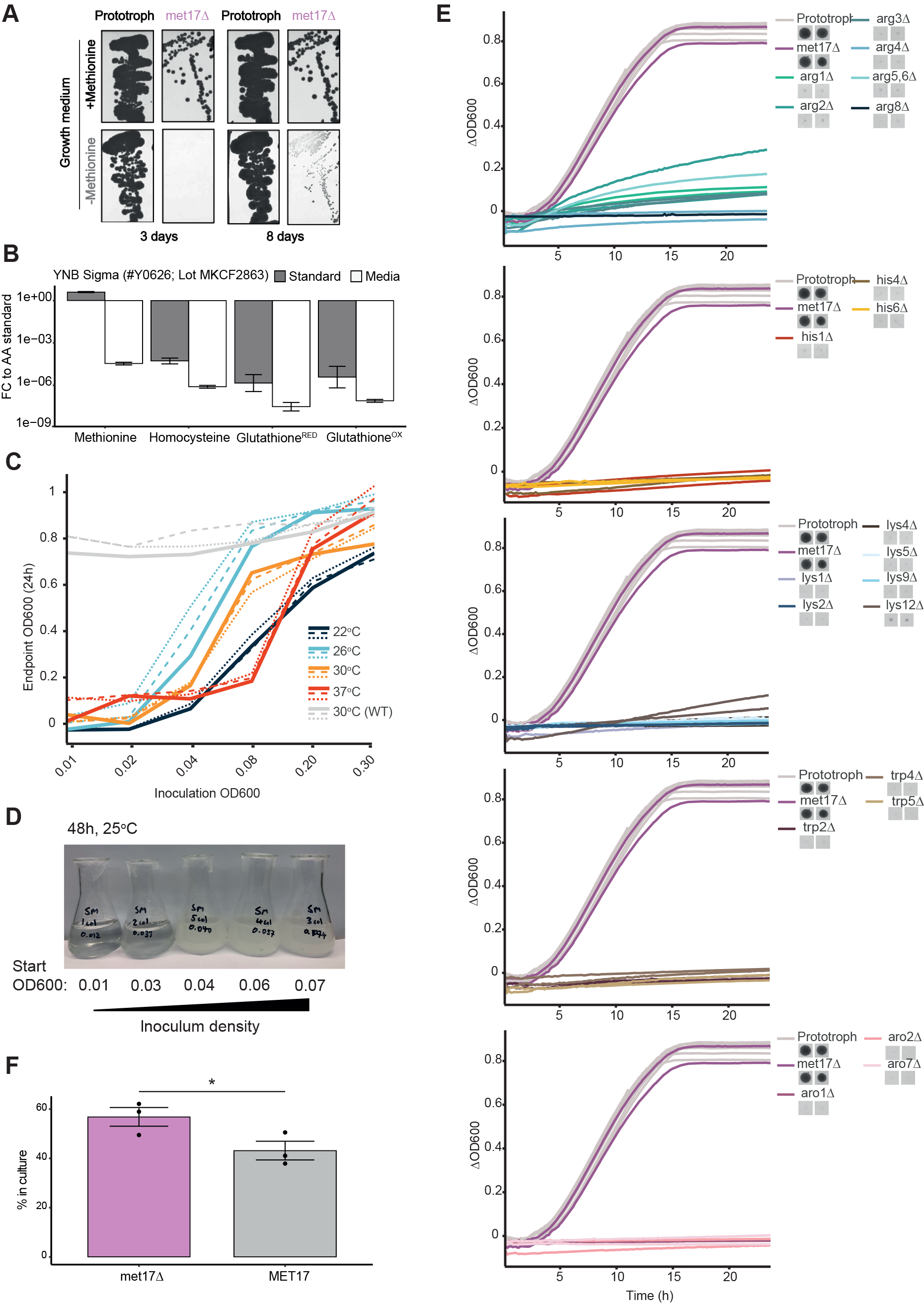

Supplement: S1 Fig — (A) Streak cultures of prototrophic WT and met17Δ strains on agar with and without methionine, imaged at 3 and 8 days incubation at 30°C. (B) LC–MS analysis of the YNB component of our minimal media. Quantified metabolites were compared to their respective standards and FC calculated. (C) Liquid cultures of met17Δ strain in minimal media without methionine inoculated at 6 different cell densities (0.01 to 0.3) and cultured at 4 different temperatures (22, 26, 30, 37°C) for 24 h. Gray indicates WT control strain that is prototrophic for methionine cultured at 30°C. Lines indicate three replicates per condition, where n = 3. (D) Growth of met17Δ strain in 20 ml cultures. Starting OD600nm of cultures varied between 0.01 and 0.07 and growth was assessed after 48 h of culture in minimal media without methionine. (E) Quantification of methionine auxotrophs in exponentially growing SeMeCo cultures. Data are mean ± SEM from 3 biologically independent replicates (n = 3). * indicates p < 0.05 via Student t test. Growth curves captured from indicated amino acid auxotrophic strains compared against the prototrophic and met17Δ strains. Inserts depict colony growth following 72 h of culture. The data underlying this figure can be found in S1 Data. FC, fold-change; LC–MS, liquid chromatography mass spectrometry; SeMeCo, self-establishing metabolically cooperating community; WT, wild-type; YNB, yeast nitrogen broth. (TIF) [file pbio.3001912.s006.tif]

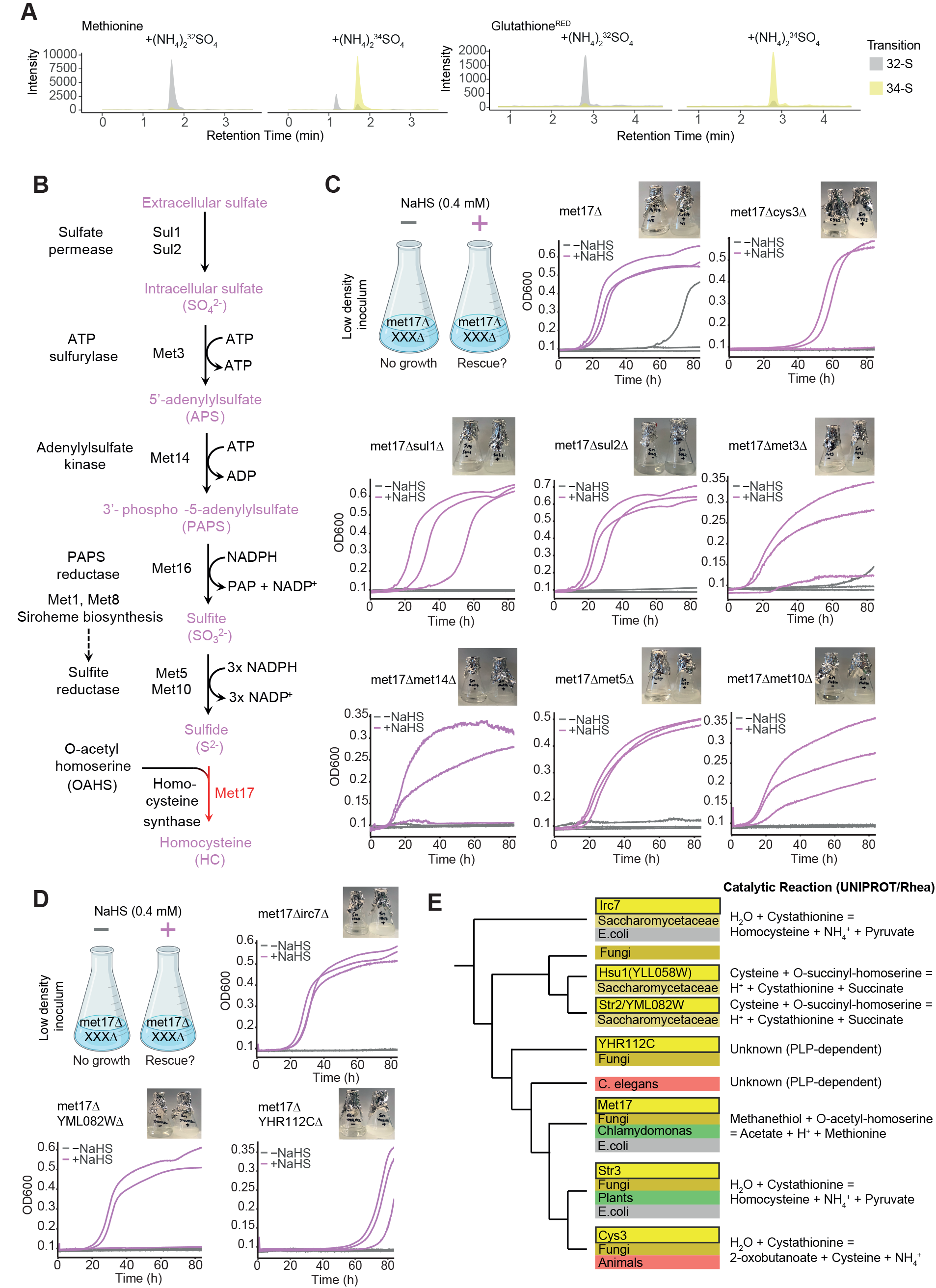

Supplement: S2 Fig — (A) Representative mass spectroscopy traces of methionine, reduced glutathione in cultures supplemented with 32S and 34S-labeled ammonium sulfate. Gray and yellow traces indicate S32 and S34 versions of the metabolite. (B) Schematic depicting the sulfur assimilation pathway from sulfate to sulfide in yeast. Metabolites carrying the sulfur are indicated in light purple. (C) Growth screens for loss of H2S utilization in sulfur assimilation deletion mutants upstream of MET17 mapped against the pathway in (B). For each strain, growth curves were captured from 3 biologically independent cultures over 84 h. (D) Screen for loss of H2S utilization in strains lacking Irc7p, YML082Wp, and YHR112Cp. For each strain, growth curves were captured from 3 biologically independent cultures over 84 h. All mutants tested are in a background of met17Δ. Scheme for Fig SB was created with BioRender.com. (E) Tree illustrating the phylogenetic relationships between orthologs of Met17p found in S. cerevisiae. After identifying orthologs in S. cerevisiae, orthologs in select model organisms were identified using BLAST and a multiple sequence alignment and tree were generated (see Materials and methods). The full tree is available in the Supporting information (S1 Data) and this summarizes the tree structure. Bold outlines indicate the S. cerevisiae proteins and other nodes represent groups of orthologs from the indicated clades. The major reaction catalyzed by each enzyme is annotated alongside as per the Uniprot/Rhea-annotated reactions database. The data underlying this figure can be found in S1 Data. (TIF) [file pbio.3001912.s007.tif]

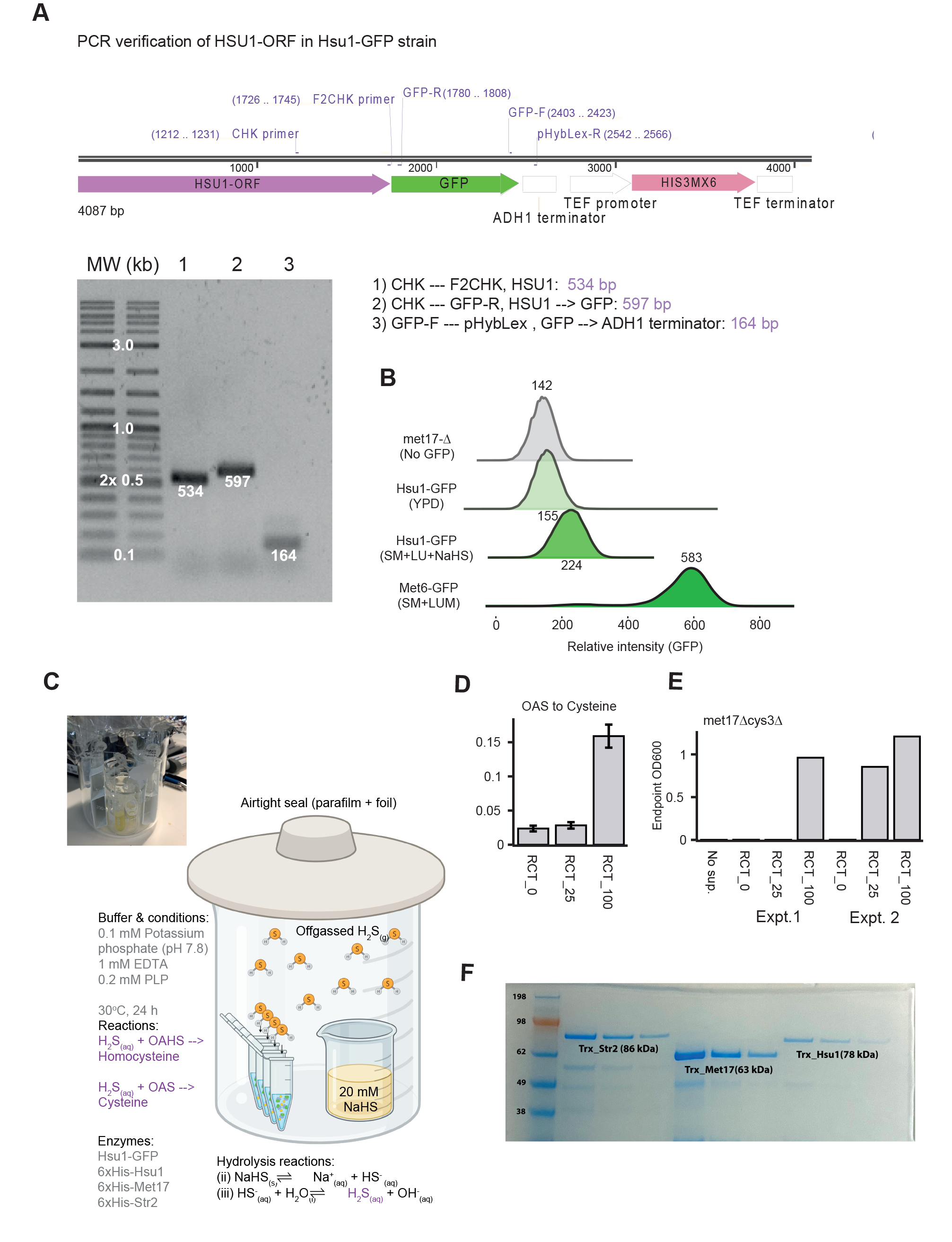

Supplement: S3 Fig — (A) PCR verification of the Hsu1-GFP ORF using genomic DNA isolated from the Hsu1-GFP strain using primers within the HSU1 and GFP open reading frames and the ADH1 terminator. All products were of the expected size as indicated by gel electrophoresis. (B) Flow cytometry analysis and confirmation of GFP intensity in response to histidine and methionine deficiency with H2S supplementation. Numbers indicate median fluorescence intensity, from approximately 40,000 events captured per strain. The met17Δ strain was used as a negative, nonfluorescent control, while the Met6p-GFP strain was used as a positive, highly fluorescent control. (C) Schematic of H2S utilization enzyme assay. Off-gassing of H2S from a 20-mM NaHS solution dissolves into reaction tubes containing either OAHS or OAS as substrate. Insert indicates actual setup of reactions. (D) Quantification of thiol concentrations via Ellman’s reagent using supernatant from enzyme assays where immunoprecipitated Hsu1-GFP was incubated with either OAS as the organic substrate. RCT_0, 25, and 100 indicate volumes of enzyme–resin slurry used in the reaction. Data are mean thiol concentrations ± SD where n = 3 biologically independent replicates. (E) Growth rescue assay of met17Δcys3Δ strain with the product of the OAS reaction. RCT_0, 25, and 100 indicate the volume of enzyme–resin complex used, which approximates increasing enzyme concentrations. OD600nm was measured from two independent sets of experiments as indicated. (F) Coomassie staining of recombinant Trx-His-tagged proteins purified via two rounds of nickel affinity chromatography. Molecular weight in kDa for the ladder and proteins are as indicated. Samples were run in triplicate in successive 1:2 dilutions. Scheme for (A) was generated with SnapGene software (www.snapgene.com). Scheme for (C) was created with BioRender.com. The data underlying this figure can be found in S1 Data. HSU1, Hydrogen Sulfide Utilizing-1; OAHS, O-acetylhomoserine; OAS, O-acetylserine [file pbio.3001912.s008.tif]
