# Supplementary material for: Inorganic sulfur fixation via a new homocysteine synthase allows yeast cells to cooperatively compensate for methionine auxotrophy
Source: PLoS Biol. 2022 Dec 1;20(12):e3001912. doi: 10.1371/journal.pbio.3001912 (PMC9757880; doi:10.1371/journal.pbio.3001912)
Supplement: Attachment — Submitted filename: 125964_1_rebuttal_2479546_rj85vk.pdf [file pbio.3001912.s009.pdf]

Full Revision

Manuscript number: RC-2022-01396
Corresponding author(s): Markus Ralser

[Please use this template only if the submitted manuscript should be considered by the affiliate journal as a full revision in response to the points raised by the reviewers.

If you wish to submit a preliminary revision with a revision plan, please use our "Revision Plan" template. ***It is important to use the appropriate template to clearly inform the editors of your intentions.****]*

1. General Statements [optional]

We thank the two reviewers for their insights and productive comments. We believe we have now generated an improved version of the manuscript that incorporates their suggestions.

2. Point-by-point description of the revisions

From Reviewer #1:

Based on the authors' data, I am quite convinced that Hsu1 is necessary for rescuing the sulfur auxotrophy of met17 mutants. However, I find the last part of the manuscript underwhelming. The authors present little direct biochemical evidence to support the proposed enzymatic activity. They used crude, immunoprecipitated material to carry out their reactions. In my opinion, all they can conclude is that Hsu1 has a role in the reactions they perform. But its function could be indirect. To properly support their conclusions, they would need to make and purify recombinant Hsu1 and then accurately draw conclusions. Otherwise, this is a case for Ephraim Racker's immortal words: "Don't waste clean thinking on dirty enzymes."

It shouldn't be too demanding to make Hsu1, perhaps a month -depending on cloning issues, and then a few days or a week to test activities in vitro. A very high confidence structure prediction from alpha-fold is also available on SGD, allowing the design of site-specific mutations to test catalytic roles.

We are glad the reviewer is convinced of the necessity of Hsu1 in rescuing the sulfur auxotrophy of met17 mutants. The reviewer asked to include an enzyme assay with a recombinantly expressed enzyme. We agree that such experiments help to exclude the possibility of a copurification artifact and would enhance our understanding of the enzyme’s catalytic function.

Therefore, we PCR amplified and cloned *MET17* and *HSU1* coding sequences from lab yeast (BY4741) genomic DNA into an *E. coli* expression plasmid containing a 6xHis-tag and Trx tag (pNH-TrxT, Savitsky et al., 2010). We then expressed and purified the recombinant protein with two rounds of NiNTA purification (SDS Page followed by Coomassie staining

(Fig. S3F)) and repeated the enzyme assay with the fusion protein instead of the immunopurified extracts. The results obtained confirmed the findings (Fig. 3E) and show that the homocysteine synthase activity of recombinant Hsu1p is comparable with Met17p (Fig. 3F).

As suggested, we have also performed sequence homology and phylogenetic analysis using structural predictions via AlphaFold,. Here we obtained a complex picture of Hsu1p as there are many related structure and sequence orthologs across entire clades of yeast. Preliminary results show that a closely related ortholog cloned from *Lachancea thermotolerans* can rescue the growth of *met17∆hsu1∆* cells, and mutagenesis of key residues alters the effectiveness of this rescue. In terms of paralogs within *S. cerevisiae*, we found that although Str2p is the enzyme bearing closest resemblance to Hsu1p, it does not appear to have homocysteine synthase activity, at least not to the degree that enables rescue of the growth defect based on our experiments (Fig. 3A, Fig. 3C). Thus, we are currently exploring whether the reverse situation could apply, i.e., whether Hsu1p might possess Str2p activity. The analysis of Str2p as well as the relationship among *HSU1* orthologues, is becoming a comprehensive study on its own, and we believe that studying the relationships among the sequence homologues goes beyond the current scope of the current study.

The statistics in Figure 1E were from two independent replicates and then four technical replicates in each case. The p-value from the rank-sum test is essentially meaningless. Better to state that the experiment was done twice (each measured four times; the technical replicates) and then report the average of each experimental replicate without any p-values.

We apologize for the poor phrasing in the figure legend. The data shown is from 2 independent experiments, each containing 4 biologically independent samples. Therefore, no technical replicates were present, and the reported p-values are valid. We have updated the figure legend to reflect this.

In Figure 2, the title says that "YLL058Wp utilizes hydrogen sulfide to rescue organosulfur auxotrophy". But the protein has not been introduced or studied yet.

We thank the Reviewer for spotting this. This was a ‘sorting error’ carried over from draft versions of the manuscript in which the order of results was different. We have now corrected this to “Hydrogen sulfide is essential for the rescue of organosulfur auxotrophy”

Page 16, the last sentence describing the data in Figure 3 reads: "Thus, YLL058Wp is necessary and sufficient for the cell to assimilate sulfide...". I agree with the "necessary", but what is the data to support the "sufficient"?

We intended for ‘sufficiency’ to refer to the ability of YLL058Wp to catalyze formation of homocysteine from OAHS and sulfide – now proven thanks to the reviewer’s suggestion of using purified, recombinant Hsu1. We have reworded the statement in the revision as follows:

"Thus, YLL058Wp is necessary for the cell to assimilate sulfide and sufficient to catalyze homocysteine biosynthesis from OAHS and sulfide."

From Reviewer #2:

The authors make a compelling case of the function of the previously uncharacterized yeast ORF YLL058W as a homocysteine synthase that can complement an MET17 deletion at high cell densities. They use mainly growth studies of deletion strains to indicate complementation, as well as yeast communities to show cooperativity and addition of alternative substrates to demonstrate the mechanisms around the function of YLL058Wp.

We thank the reviewer for their positive assessment of our work.

As this is a major point of your investigation, a clear definition of what you regard as high cell density is needed. At one point you call OD 0.02-0.3 high-density, at another OD 0.02-0.03 are regarded as low-density, and at a third you define high-density as OD 0.08. A clear statement of your definition would be appreciated. Also, on p. 6 you refer to inoculum sizes below OD 0.01, but you show no data in that range.

We apologize for the imprecise language and agree with the reviewer that low- and high-density inoculums require clear definition. We have now used the results of the temperature vs inoculation OD experiment (Fig. 1B) as a basis and defined low and high inoculums as $OD_{600nm} \leq 0.01$ or $OD_{600nm} > 0.02$ respectively.

I disagree with figure 1D and the following conclusions. I can agree that met*17*Δ exhibits growth that is close to WT and that some other deletion strains are not growing. BUT I do not believe you can state the conclusion you have with that strong confidence, mainly because you do not show the same time span for all growth experiments. When characterizing met*17*Δ you have always chosen to report the OD values of either 24 or 48 h, while for the deletion screen you choose to show full growth curves but only up to 20 h. What happens thereafter is not known but I don't find it impossible that at least several of the deletion strains would reach OD's similar to WT or *met17*Δ. Considering this, I would urge you to revise the wording around these results on p. 6. I also find the sentence "All strains except *met17*Δ were growth deficient, despite high-density inoculation and the absence of amino acid supplementation." unclear. Are the strains really growth deficient despite the absence of amino acid supplementation?

The reviewer has a valid point. We studied the growth of these mutants more closely and noticed that media carry over explains a residual growth phenotype in several of the methionine pathway mutants. This phenotype is however largely abolished when we revive the strains from cryostock onto solid synthetic complete media, allow them to grow out for 3 days, and then directly inoculate liquid synthetic minimal media (thus avoiding any carryover). In both a growth curve (Fig. 1C, Fig. S1E) and a plate assay, we find that only *met17*Δ yeast have a significant growth rate in the media lacking an organic sulfur source.

This result is now fully consistent with the literature. The growth assays have been replaced in the manuscript.

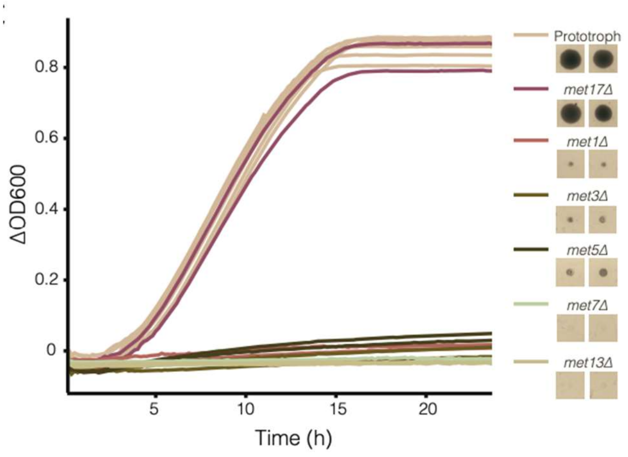

Revision Figure 1 *Growth assay with improved protocol to remove media carryover (Fig. 1C in current manuscript). Among the methionine auxotrophic gene deletion strains, only the met17Δ strain reaches a significant growth rate.*

In the process of redesigning and redoing the growth experiments, we also generated custom media to test whether sulfur limitation is a condition under which Hsu1p activity can confer a growth advantage that allows it to be conserved. We find that *HSU1* mutants grow slower under sulfur limitation. These findings strengthen our results and have been added to a new Figure 4.

The last couple of sentences of both the Results and the Discussion seem disjointed from the overall discussion. I would not draw any conclusions regarding the function of YLL058W from only the phenotype of a *met17Δcys3Δ* strain, and in general the last paragraph of the Results read more like Discussion. The last paragraph of the Discussion seems ok content wise but the text would feel more rounded out if you were to include your present results in the discussion of microbial communities.

We agree with the reviewer and have removed these discussion points as they were too speculative.

The authors are inconsistent when it comes to statistical analysis. Only two graphs include any statistics, and two different statistical tests are used without justification. Tests could also have been performed for the data in figs 1F, 1H, 2E, 3E, 3F, S1A, S1E, S3D at least, and probably for some of the growth-based assays.

As suggested by the reviewer, we have now reviewed and adapted the use of statistical tests for all data. Specifically, for Fig. 1E (formerly Fig. 1F), a two-sided Wilcoxon Rank Sum test was performed. Fig. 1H was removed. For figures 2E, 3E, 3F, 3G (formerly Fig. 3F), S1B, S1F (formerly S1A and S1E respectively) and S3D a two-tailed Student's t-test was performed between the indicated samples. We elected to present all growth curves with the

independent replicates overlaid. As most of these indicate growth versus no growth and show all replicates, we deem it unnecessary to include statistics.

I do not believe any new experiments are needed to address my main comments. However, I would suggest significant re-working of all the figures to increase readability and accessibility, and a slight re-write of the manuscript to modulate the claims to fit the actual strength of the data better.

We appreciate the support and input of the reviewer, and we hope that we have been able to fully address their comments. The manuscript has been reworked for clarity and simplicity of the text.

Minor comments:
Figures are crowded and hard to read, especially metabolic maps. Why are colors used in fig 1A, 2B, 2F, S2B, S2C if they are not explained? The changes in color use between figures are also confusing. I think one really good, elaborate, figure of your understanding of the metabolic relationships would be more impactful than the four slightly different ones you show and want the reader to compare and contrast. The color choices could also be improved, especially for accessibility (e.g. using red, pink and purple in the same schematic).

We agree and have now simplified the color usage metabolic maps to better highlight the relationships between enzymes and metabolites. The major metabolites that contain sulfur are now consistently highlighted in light purple in all pathway maps. Fig. 1A presents the major core of the methionine/cysteine biosynthetic pathway, with associated pathways that feed into it highlighted in different colors that correspond to enzyme expression changes subsequently highlighted in Fig.1F. Fig. 2B is not a metabolic map, but rather a summary of the hydrolysis reactions of sulfide that occur within and outside the cell and upon supplementation with the sulfide donor NaHS. We have also highlighted the sulfur-bearing chemical species here in light purple to maintain consistency. Similarly, Fig. 2F both simplifies and extends the metabolic map relating to methionine/cysteine biosynthesis and hydrogen sulfide overflow respectively and nicely summarizes the path of sulfur in the isotopic tracing experiment. Fig S2B delineates the entire sulfur assimilation pathway in full (abbreviated in Fig. 1A) and aids interpretation of the growth curves adjacent in Fig. S2C. We have also removed the former Fig. S2C as it is described elsewhere (Fig. 3B). The color scheme in Fig. 3B has also been greatly simplified and the enzyme names removed to improve clarity.

I would strongly advise you to consider replacing photographs of shake flasks with the corresponding OD readings. It will save you space for figures and be much easier for the reader to appreciate the growth. Several times I thought "Well, if you say so... " when assessing growth in these photographs, and that is not the way to convince a reader. Keep photographs only if they add information a numerical value cannot, such as for the precipitation assay.

We understand the reviewer's point that visual depiction of shake flasks in many instances does not add anything to the interpretation and can be substituted with OD readings. There are other instances in our manuscript where it could be hard for the reader to judge the experimental setup from the text or OD value alone, i.e., the precipitation assay (Fig. 2A) and the connection of two flasks by rubber tubing (Fig. 3C). In order to address the Reviewer's valid point, we have now chosen a 'hybrid' format. The data is presented mainly as growth curves, with shake flask photos used as a visual representation of the growth endpoint.

Of note, we thank the reader for this recommendation as the growth curves also revealed a delayed growth phenotype in certain mutants *(met17*Δ*str2*Δ and *met17*Δ*str3*Δ*)* that adds to the evidence pointing to the existence of a bypass enzyme.

You mention larger scale cultures on p. 6 (with ref to S1C) but no mention of the scale of previous cultures.

We apologize for this oversight, the scale of the previous cultures refers to a 96-well format. We have now amended this in the manuscript.

Figure 1B does not convince me that a higher inoculum OD yields growth of met17delta strains, and I don't really understand why you've chosen to include it.

This figure was included because previous investigations of this phenomenon were observed when cells were streaked at high density on solid media in the absence of methionine. Hence, the analysis shows that we reproduce previous findings with our own strain, and with more minimal media formulations that are better suited fo the study of metabolism. We however agree with the reviewer that this is not necessary to show in the main figure and since all of the subsequent experiments were conducted using liquid cultures, we have now moved this into the supplementary data (Fig. S1A).

To me figures 1G and 1H are redundant considering how you discuss them.

This is a valid point. We have now removed Fig. 1H.

You talk a lot about *MET17* homologs, but it's hard to keep track of how many and what they are. A table, potentially including experimental results for these genes might help the reader follow your reasoning.

This is also a valid point; we have constructed a table summarizing the functional annotation and results for all the homologs in the revision as suggested (Table S2), and included a phylogeny of the *MET17* homologs (Fig. S2E).

The choice of what methionine cycle enzymes to highlight in the proteomics analysis appears to me like cherry-picking. For example, Sah1p and Ser3p that are also described as core enzymes but show lower abundance, which I believe is worth mentioning.

Review COMMONS

Our aim was not just to describe methionine cycle enzymes that are upregulated, but rather that there are changes in protein levels throughout the entire pathway that might indicate rerouting/reconfiguration of metabolism, either due to *met17Δ* itself or because of bypass activation. We have now mentioned the lower abundance observed as suggested and toned down the wording to reflect that fact that not all implicated enzymes show increased abundance.

Did you check where in the genome the GFP-fused YLL058W ORF was integrated? Did you sequence the ORF? It looks like you only checked if the insert existed. Being sure the gene is expressed under the correct promoter is important to your conclusions.

The strain was sourced from the work of Huh et al. 2003, where the GFP coding sequence was designed for targeted insertion downstream of the genes of interest. The fusion protein is under the control of the gene's native promoter. We validated the strain via PCR to ensure that we were using the correct strain and have made this reason clear in the revised main text.

You claim on p. 21 that your data indicates *HSU1* expression (genes are expressed, not proteins) responds to high sulfide levels. What specific experimental result are you referring to?

The sentence referred to the increase in protein levels which we observed in Fig. S3B whereby conditions were optimized for Hsu1-GFP immunoprecipitation. We found that the absence of methionine together with the supplementation of NaHS (i.e., high sulfide) increases the levels of Hsu1-GFP fusion protein. We additionally find that levels of Hsu1p are also increased upon sulfur limitation (Fig. 4B). This is in line with a previous transcriptomic study of sulfur limitation, where *HSU1* is upregulated, but the study did not pursue this observation further (Fig. 4A). Together, this suggests that levels of Hsu1p are responsive to both sulfur limitation and the presence of high sulfide. As aforementioned, we have now also evidence that *HSU1* deletion strains have a moderate but significant growth defect under sulfur limitation and have included these new results as Figure 4. We hope these edits make the point clearer.

There are several spelling and grammatical mistakes throughout the text. This includes breaking naming conventions of genes and proteins (e.g., calling a gene YLL058Wp in the abstract, a protein (?) Met17 in the end of the discussion and inconsistencies in the camel-case of Hydrogen Sulfide Utilizing-1).

Thanks for pointing these issues out, we rectified these mistakes and others in the main text.

I believe the Abstract would be more effective if you included some of the methodology (such as the SeMeCo experiment) used throughout the study.

We agree with the reviewer and have mentioned the use of SeMeCo in the abstract.

There seems to be some issues with your reference management system. The second reference to Cost & Boeke (The Met15 Resource, 1999) seems to be alphabetized according to first name rather than last. Also, in the text Boeke is not recognized as an author, which makes a citation made on the top of p. 7 especially confusing. You also have some "dead" citations that just appear as numbers (mid p. 11 and 14), and several times your references to figures do not correlate with what is actually shown (at least figures 1B on p. 5; 2A, 2E, S2B on p. 12).

We apologize for this oversight. These were carryovers from several rounds of revision of the draft manuscript and had escaped our attention. All references and citations have now been corrected.

You could be clearer in Materials and Methods. Mention recipes in the Yeast culture section, not later. u is used instead of the micro symbol sometimes. Under the isotropic tracing section, you say that "Samples were essentially treated as described above" without indication of what type of previous experiment you are referring to. Under Hydrogen sulfide utilization enzyme and growth assays a setup for NaHS diffusion is described, and I assume the Eppendorf tubes used would have to be open, but there is no mention of this. If my assumption is incorrect, please explain how the assay works with closed tubes.

We apologize those certain parts of the materials and methods are unclear and have now corrected all the above. Specifically, the reviewer's assumption is correct – since the enzyme assay relies on the diffusion of gaseous H_2S the Eppendorf tubes are indeed left open within the larger sealed reaction vessel that prevents its escape from the system (see insert photo, Fig. S3C).

Please include the general background strains for table S1 (e.g. CEN.PK, S288c), especially since you use strains from multiple sources and you discuss S288c in the end of the Results section.

We have included the background information of all strains used in this study (Table S1).
