# Supplementary material for: Inorganic sulfur fixation via a new homocysteine synthase allows yeast cells to cooperatively compensate for methionine auxotrophy
Source: PLoS Biol. 2022 Dec 1;20(12):e3001912. doi: 10.1371/journal.pbio.3001912 (PMC9757880; doi:10.1371/journal.pbio.3001912)
Supplement: Attachment — Submitted filename: Response to Editors.docx [file pbio.3001912.s011.docx]

**Response to Editor’s Comments**

Thank you for your patience while we considered your revised Review Commons manuscript "Inorganic sulfur fixation via a new homocysteine synthase allows yeast cells to cooperatively compensate for methionine auxotrophy" for publication as a Research Article at PLOS Biology. This revised version of your manuscript has been evaluated by the PLOS Biology editors, the Academic Editor, and one of the original reviewers.

Based on the review and on our Academic Editor's assessment of your revision, we are likely to accept this manuscript for publication, provided you satisfactorily address the following data and other policy-related requests.

IMPORTANT: Please attend to the following:

a) We are aware of the closely related paper from the Carvunis lab, whose preprint you cite in your paper. The Academic Editor was concerned to minimize confusion in the field around nomenclature and wondered if you could possibly liaise with Carvunis and colleagues to try to ensure harmonization over "Hsu1"/YLL058W (I see that their preprint sticks with the latter, but this may have changed since initial deposition).

*We too have noticed that the Carvunis lab attributed a similar enzymatic function to Hsu1/YLL058W that parallels our study and also published their findings as a preprint. We are glad that the results are largely consistent, despite the different approaches and experiments that led to the discovery of the enzyme. We believe this provides greater confidence in the quality of both studies, and more importantly, independent replication that all but confirms Hsu1/YLL058W as a bona fide homocysteine synthase that rescues methionine auxotrophy in the absence of Met17.*

*To avoid confusion with the gene name, we have followed the guidelines of the yeast community, and reserved ‘HSU1’ as a gene symbol for YLL058W (which is the systematic name that serves as coordinates to the actual ORF). Therefore, we see no conflict in the use of both names as this is a common and accepted convention in the field.*

*On the topic of gene name, we made this reservation on the 9th September 2021 before any of the preprints were published online, and were the first in reserving a gene symbol for YLL058W (see enclosed email). In agreement with the Gene Name Reservation procedure outlined by the biocurators of the Saccharomyces Genome Database (SGD), upon the publication of our manuscript (or publication of another manuscript that refers to YLL058W as ‘HSU1’), HSU1 will immediately become the standardized gene name and will be linked to the database* [*https://www.yeastgenome.org/locus/S000003981*](https://www.yeastgenome.org/locus/S000003981)*. Indeed, it has also not escaped our and the curator’s attention that the secondary condition has already been met* [*https://pubmed.ncbi.nlm.nih.gov/35690444/*](https://pubmed.ncbi.nlm.nih.gov/35690444/)*. Therefore, convention dictates that HSU1 is already the official gene name alongside the systematic designator of YLL058W.*

b) Still on the topic of the Carvunis study, I see that it's listed as ref 41 in your bibliography, but there's no in-text citation to it; please could you rectify this? I couldn't find a citation for ref 40, either. If you do contact Carvunis, it may be helpful to obtain a more up-to-date citation ("in press" at a specific journal, rather than the preprint); if it publishes soon, you could also update your paper at the proof stage.

*We apologize for this oversight. We noted the preprint during our revisions and thus had added the reference during the revision process. We have added the following statement to the manuscript: “While this manuscript was in the peer review process, a parallel study was published as a preprint* [*(41)*](https://paperpile.com/c/tZwoGU/IueEQ)*. Although both studies use different approaches, they agree on the enzymatic function of Hsu1 and the involvement of H_2_S as a substrate.” We have also removed the original ref 40 as the statement it supported was removed during the last revision.*

c) Please provide a blurb, according to the instructions in the submission form.

*We have provided a blurb as requested: In yeast, Met17p deletion induces the overflow of hydrosulfide ions due to methionine auxotrophy. Yu et al., report the discovery of a new H_2_S utilizing enzyme, Hsu1p that recycles hydrosulfide and confers a growth advantage under sulfur limitation. This enzyme allows the cells to overcome methionine auxotrophy when grown in high density, underscoring the importance of cell-cell metabolic interactions.*

d) Please address my Data Policy requests below; specifically, we need you to supply the numerical values underlying Figs 1BCDEF, 2E, 3AEFGH, 4BCE, S1BCEF, S2ACD, S3BDE, either as a supplementary data file(s) or as a permanent DOI’d deposition.

*All raw data from which the listed figures were generated have now been included as ‘Supplementary Data file 1’, with specific reference to the corresponding sub-figures within. We have not included the raw data for Fig.1E and 1F since this has already been previously deposited in the PRIDE database with the dataset identifier PXD031160.*

e) Please cite the location of the data clearly in all relevant main and supplementary Figure legends, e.g. “The data underlying this Figure can be found in S1 Data” or “The data underlying this Figure can be found in <https://doi.org/XXXX>”

*We have now added this statement to all relevant main and supplementary figures. The exception to this case is data for Fig. 1E and F, which represents a reanalysis of existing proteomics data deposited in the PRIDE database as mentioned above. We have however included a statement to make this clear in the Fig. 1 legend.*

As you address these items, please take this last chance to review your reference list to ensure that it is complete and correct. If you have cited papers that have been retracted, please include the rationale for doing so in the manuscript text, or remove these references and replace them with relevant current references. Any changes to the reference list should be mentioned in the cover letter that accompanies your revised manuscript.

*We have now reviewed our reference list and ensured that it is now relevant, complete and correct. We did not identify any papers that were retracted and have summarized all changes in the accompanying cover letter.*
